# Paracentral acute middle maculopathy in patient with atherosclerosis and angioplasty with stents in the coronary artery

**DOI:** 10.31744/einstein_journal/2021RC5521

**Published:** 2021-02-19

**Authors:** Diogo Gonçalves dos Santos Martins, Ever Ernesto Caso Rodriguez, Yandely Chihuantito Choquechambi, Thiago Gonçalves dos Santos Martins

**Affiliations:** 1 Hospital dos Servidores do Estado do Rio de JaneiroRio de JaneiroRJBrazil Hospital dos Servidores do Estado do Rio de Janeiro , Rio de Janeiro , RJ , Brazil .; 2 Universidade de São PauloSão PauloSPBrazil Universidade de São Paulo , São Paulo , SP , Brazil .; 3 Instituto Suel AbujamraSão PauloSPBrazil Instituto Suel Abujamra , São Paulo , SP , Brazil .; 4 Universidade Federal de São PauloSão PauloSPBrazil Universidade Federal de São Paulo , São Paulo , SP , Brazil .

**Keywords:** Macula lutea, Retinal vessels, Constriction, pathologic, Tomography, optical coherence, Macular degeneration, Angioplasty, Stents

## Abstract

Sophisticated imaging systems have helped to redefine the clinical presentation of acute macular neuroretinopathy and have markedly enhanced diagnostic sensitivity. The proposed mechanism of paracentral acute middle maculopathy is related to ischemia at the level of the superficial and deep retinal capillary plexi. This is a case report of a patient who developed an acute macular neuroretinopathy after an uneventful angioplasty with stents in the coronary artery.

## INTRODUCTION

The proposed mechanism of paracentral acute middle maculopathy (PAMM) is related to ischemia at the level of the superficial and deep retinal capillary plexi. ^( 1 )^ However, more recently, this manifestation has been described in association with other conditions of ischemic etiology. ^( 2 )^ We describe a rare case of acute macular neuroretinopathy (AMN) in a patient immediately after the angioplasty with stents in the coronary artery.

## CASE REPORT

A 56-year-old white female sought to ophthalmic care in January 2016 complaining to paracentral scotoma in the left eye for 1 week. She noticed the onset of the symptom 1 day after an uneventful angioplasty with stents in the coronary artery after a chronic coronary insufficiency. As comorbidities she had systemic arterial hypertension and atherosclerosis. There was no report on the use of sympathomimetic medication in this patient, and no history of recent flu-like diseases. The patient denied the use of caffeine and oral contraceptives. There were no data from the patient’s carotid doppler examination. The best-corrected visual acuity (BCVA) was 20/20 on the right eye and 20/40 on the left eye. Amsler grid test showed a small superior paracentral scotoma in the left eye. Biomicroscopy of the anterior segment was normal.

**Figure 1:**
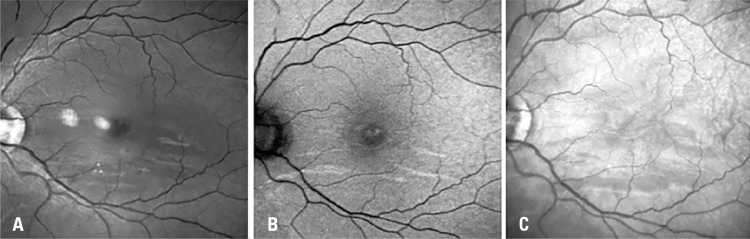
Red-free photography, blue-light autofluorescence and near infrared reflectance in the Spectralis ® HRA + optical coherence tomography (A, B and C, respectively), obtained after 7 days of symptom onset. Near infrared reflectance and red-free photography images show paracentral dark grey wedge-shaped lesions and bands affecting the temporal area and the area below the fovea of the left eye. Blue-light autofluorescence images show hyper-autofluorescence bands below the fovea

**Figure 2:**
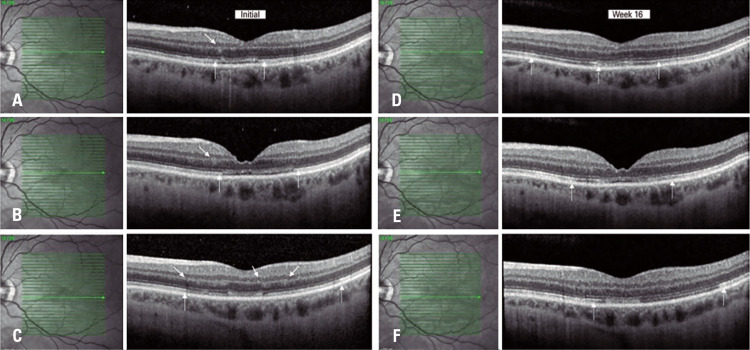
Optical coherence tomography images showed a hyperreflective band on the inner nuclear layer and outer plexiform layer. There is also attenuation of the underlying retinal pigment epithelium consistent with a type 2 lesion (A, B and C). After 16 weeks, we noted a thinning of the inner nuclear layer and the disappearance of the hyperreflective area (D, E and F)

Retina examination showed a parafoveal wedge-shaped lesion seen in the red-free and bands with hyper-autofluorescence on blue-light autofluorescence on the left eye (Figures 1A and 1B). No alteration was found on the right eye. Fluorescein angiography was normal. Near infrared reflectance (NIR) of the left eye demonstrated multiple dark gray wedge-shaped lesions and paracentral lesions (Figure 1C). The optical coherence tomography (OCT) of the lesion revealed a hyperreflective band at the level of the inner nuclear layer (INL) and outer plexiform layer (OPL) with attenuation of the underlying inner segment/ outer segment (IS/OS), and OS/retinal pigment epithelium layers ( Figure 2 ).

**Figure 3:**
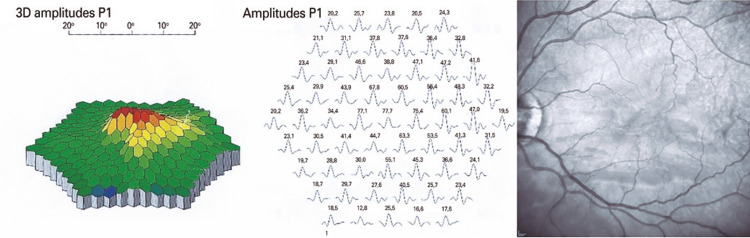
Multifocal electroretinogram showing the reduction of the amplitude of the waves in the central and paracentral area

After 4 months the patient’s BCVA remained 20/40 on the left eye with persistence of the scotoma. Near infrared reflectance confirmed persistence of the lesion, although the lesion appeared unchanged in comparison with the baseline examination ( Figure 2 ). The OCT revealed that the hyperreflective area was resolved with subsequent thinning of the INL (between top solid arrows) overlying an attenuated IS/OS and OS/retinal pigment epithelium complex (Figures 2D to 2F). The multifocal electroretinogram (mfERG) demonstrated a reduction of amplitude of waves in the central and paracentral area of the fovea ( Figure 3 ).

## DISCUSSION

Paracentral acute middle maculopathy was initially described as an ischemic variant of the AMN. When identifying PAMM, it was described as an unilateral condition whereas AMN has been described as either as unilateral or bilateral. Two subtypes of PAMM have been observed: type 1 is located in the OPL and INIL, whereas type 2 is deeper and involves the OPL and the retinal pigment epithelium. ^( 1 )^ Both types result in a paracentral scotoma. ^( 1 )^ Type 1 has been described more recently whereas type 2 is consistent with previous reports of AMN. ^( 3 )^

Paracentral acute middle maculopathy is a deep retinal circulation ischemia, as demonstrated by current evidence ^( 2 , 4 , 5 )^ that found that the vessel density of the deep capillary plexus was reduced when compared with the normal fellow eye (-19.4% reduction, p=0.04). ^( 4 )^

Paracentral acute middle maculopathy has been reported in association with other retinal vascular diseases such as diabetic and hypertensive retinopathy, retinal artery occlusion, central retinal vein occlusion, sickle cell retinopathy, and Purtscher retinopathy. ^( 6 - 10 )^

We believe this case report is consistent with type 2 AMN involving the deep retinal plexus in association with coronary disease. We could not confirm with certainty the etiology of vascular obstruction. Given that PAMM is a recent described entity; its association with coronary disease is possible to be underrecognized. This report documentation occurred in 2016 when the angiography OCT exam was very restricted in Brazil, and for this reason this exam was not performed. Further studies are needed to better determine the incidence of the PAMM associated with coronary artery disease. Clinicians should be especially aware of this condition, mainly in patients with new paracentral visual scotoma, given the subtle changes in the fundoscopy seen in PAMM, because this manifestation may be observed in a variety of clinical scenarios.
