# Supplementary material for: Paracentral acute middle maculopathy in patient with atherosclerosis and angioplasty with stents in the coronary artery
Source: Einstein (Sao Paulo). 2021 Feb 19;19:eRC5521. doi: 10.31744/einstein_journal/2021RC5521 (PMC7901525; doi:10.31744/einstein_journal/2021RC5521)
Supplement: Translation

# Translation: Maculopatia média aguda paracentral em paciente com arterioesclerose e angioplastia com *stent* na artéria coronária

**Authors:** Diogo Gonçalves dos Santos Martins, Ever Ernesto Caso Rodriguez, Yandely Chihuantito Choquechambi, Thiago Gonçalves dos Santos Martins

**Affiliations:** 1 Brasil Hospital dos Servidores do Estado do Rio de Janeiro, Rio de Janeiro, RJ, Brasil .; 2 Brasil Universidade de São Paulo, São Paulo, SP, Brasil .; 3 Brasil Instituto Suel Abujamra, São Paulo, SP, Brasil .; 4 Brasil Universidade Federal de São Paulo, São Paulo, SP, Brasil .

**Keywords:** Macula lutea, Vasos retinianos, Constrição patológica, Tomografia de coerência óptica, Degeneração macular, Angioplastia, Stents

## Abstract

Sistemas de imagem sofisticados ajudaram a redefinir a apresentação clínica da neurorretinopatia macular aguda e têm sensibilidade diagnóstica marcadamente aumentada. A maculopatia média aguda paracentral tem sido relacionada à isquemia ao nível dos plexos capilares superficial e profundo da retina. Este é um relato de caso de paciente que desenvolveu uma neurorretinopatia macular aguda após uma cirurgia de angioplastia com *stents* da artéria coronária sem complicações.

## INTRODUÇÃO

O mecanismo proposto de maculopatia média aguda paracentral (MMAP) é relacionado à isquemia no nível superficial e dos plexos capilares superficiais. ^( 1 )^ Porém, recentemente, tem sido descrita a associação com outras condições de etiologia isquêmica. ^( 2 )^ Relatamos um caso raro de neurorretinopatia macular aguda (NMA) em paciente logo após ser submetido à angioplastia com *stents* em artéria coronária.

## RELATO DE CASO

Paciente do sexo feminino com 56 anos que procurou serviço oftalmológico em janeiro de 2016 mencionando escotoma paracentral em olho esquerdo por 1 semana. A paciente relatou início dos sintomas 1 dia após realizar angioplastia sem intercorrências com *stent* em artéria coronária, após episódio de insuficiência cardíaca crônica. Em relação a comorbidades, ela apresentava hipertensão arterial sistêmica e aterosclerose. Além disso, nessa paciente, relatou-se o uso de droga simpaticomimética, e não havia histórico recente de doença. A paciente negou história recente de diagnóstico de influenza e uso de cafeína e contraceptivos. Não se observaram achados no exame Doppler de carótidas da paciente. A visão de melhor acuidade visual corrigida (MAVC) foi 20/20 em olho direito e 20/40 em olho esquerdo. O teste com a tela de Amsler mostrou um escotoma paracentral superior em olho esquerdo. A biomicroscopia de segmento anterior estava normal.

O exame de retina mostrou lesão nas margens da área perifoveal observada na *red-free* e bandas com hiperautofluorescência em autofluorescencia de luz azul no olho esquerdo (Figuras 1A e 1B). Não foi encontrada alteração no olho direito. A angiografia fluoresceínica estava normal. A reflectância do infravermelho próximo (RIP) do olho esquerdo monstrou múltiplas lesões cinza-escuras em formato de cunha e lesões paracentrais (Figura 1C). A tomografia de coêrencia óptica (OCT) da lesão revelou uma banda hiperreflexiva no nível da camada nuclear interna (CNI) e camada plexiforme externa (CPE), com atenuação de segmento interno/externo subjacente (SI/SE) e camada do SE/epitélio pigmentar da retina (EPR) ( Figura 2 ).

**Figura 2:** Imagens da tomografia de coerência óptica mostrando banda hiperreflexiva na camada nuclear interna e camada plexiforme externa. Observa-se também atenuação do epitélio pigmentar da retina com lesão tipo 2 (A, B e C). Após 16 semanas, notaram-se uma fina camada nuclear interna e o desaparecimento da área hiperrefletida (D, E e F)

Após 4 meses, a paciente com MAVC permaneceu com 20/40 em olho esquerdo e escotoma persistente. A RIP próxima confirmou persistência da lesão, apesar de parecer inalterada em comparação com exame de linha de base ( Figura 2 ). A OCT revelou que a área de hiperreflexão foi resolvida com subsequente afinalamento da CNI (entre as setas sólidas no topo) subjacente a SI/SE atenuado e SE/EPR complexo (Figuras 2D a 2F). O eletrorretinograma multifocal (mfERG) mostrou redução da amplitude das ondas na área central e paracentral da fóvea ( Figura 3 ).

**Figura 3:** Eletrorretinograma multifocal mostrando redução da amplitude das ondas na área central e paracentral

## DISCUSSÃO

A MMAP foi inicalmente descrita como variante isquêmica da NMA. A MMAP é descrita como condição unilateral, enquanto a NMA é detalhada tanto como unilateral quanto como bilateral. Dois subtipos de MMAP têm sido observados: tipo 1, localizado na CPE e CNI, e tipo 2, que é profunda e envolve a CPE e a EPR. ^( 1 )^ Ambos os tipos resultam em escotoma paracentral. ^( 1 )^ O tipo 1 tem sido descrito mais recentemente, enquanto o 2 é consistente com relatos anteriores de NMA. ^( 3 )^

A MMAP é uma oclusão venosa isquêmica da retina, como exposto em evidências atuais, ^( 2 , 4 , 5 )^ que demonstram que a densidade das veias do plexo capilar profundo foi reduzida quando comparada a do outro olho normal (-19,4% de redução, p=0,04). ^( 4 )^

A MMAP tem sido descrita em associação com outras doenças vasculares da retina, como diabetes, retinopatia hipertensiva, oclusão arterial retiniana, retinopatia de anemia falciforme e retinopatia de Purtscher. ^( 6 - 10 )^

Acredita-se que o caso relatado é consistente com tipo 2 da NMA, envolvendo o plexo capilar profundo da retina em associação com doença coronária. Não foi possível confirmar com acurácia a etiologia da obstrução vascular. Considerando que MMAP é uma entidade recentemente descrita, é possível que essa associação seja sub-reconhecida. A documentação deste caso ocorreu em 2016, quando a angiografia por coerência óptica era muito restrita no Brasil, não sendo realizada por tal motivo. Outros estudos são necessários para compreender melhor a incidência da MMAP associada com a doença arterial coronariana. Porém, em pacientes com escotoma paracentral visual, os médicos clínicos devem ter especial conscientização sobre essa condição, considerando que mudanças sutis na fundoscopia são observadas na MMAP e podem ser apresentadas em uma variedade de cenários clínicos.

**Figura 1:** Imagens *red-free* , autofluorescencia de luz azul e reflectância do infravermelho próximo no Apectralis ® HRA + tomografia de coerência óptica (A, B e C, respectivamente) obtidas após 7 dias do início dos sintomas. A reflectância do infravermelho próximo e as imagens fotográficas *red-free* mostram lesões paracentrais com cinza-escuro em formato de cunha e bandas afetando a área temporal e a área abaixo da fóvea do olho esquerdo. Imagens em autofluorescencia de luz azul mostram bandas de hiperautofluorescência abaixo da fóvea
